# Emergence of *bla*_TEM_ Type Extended-Spectrum **β**-Lactamase Producing *Salmonella* spp. in the Urban Area of Bangladesh

**DOI:** 10.1155/2014/715310

**Published:** 2014-03-10

**Authors:** Dilruba Ahmed, Abu Iftiaf Md. Salah Ud-Din, Syeda Umme Habiba Wahid, Razib Mazumder, Kamrun Nahar, Anowar Hossain

**Affiliations:** International Centre for Diarrheal Disease Research, Bangladesh (icddr,b), Dhaka 1212, Bangladesh

## Abstract

Salmonellosis, an acute invasive enteric infection, is endemic in Bangladesh. We analyzed 128,312 stool samples of diarrheal patients to identify *Salmonella* spp. during 2005–2013. A total of 2120 (1.7%) *Salmonella* spp. were isolated and the prevalence of *Salmonella* spp. decreased significantly over time (2→1%, *P* < 0.001). Among the typhoidal *Salmonella* (TS) serogroups, *S.* Typhi was predominant (404, [65.1%]) followed by *S.* Paratyphi B (139, [22.4%]) and *S.* Paratyphi A (78, [12.6%]). Of the nontyphoidal *Salmonella* (NTS) isolates, the serogroup C1 (560, [37%]) was predominant followed by B (379, [25%]), C2 (203, [14%]), E (127, [9%]), and D (94, [6%]). Most of the resistance was found towards nalidixic acid (40%), ampicillin (36%), cotrimoxazole (20%), chloramphenicol (13%), ciprofloxacin (4%), and ceftriaxone (4%). Interestingly, 32% of the isolates showed reduced susceptibility to Cip. Multiantibiotic resistance (MAR, ≥3 drugs) was more common among TS than NTS strains (*P* < 0.001). Among the representative ceftriaxone-resistant isolates, *bla*_TEM_ gene was detected among 88% (7/8) of the strains, whereas only one strain of *S.* Typhi was positive for both *bla*_TEM_ and *bla*_CTX-M_ genes. The study reflects higher prevalence of MAR *Salmonella* spp. and is the first to report the *bla*_TEM_ gene mediated ESBL production among *Salmonellae* in Bangladesh. Emergence of MAR *Salmonella* spp. in particular ESBL strains should be considered a public health concern.

## 1. Introduction

Salmonellosis due to nontyphoidal* Salmonella* (NTS) infection is a global public health concern, particularly in* Salmonella* endemic low and middle income countries (LMICs). Treatment is critical for persons with severe disease, particularly children and immune compromised people. Treatment with an appropriate antibiotic can shorten the duration of symptoms, significantly reduce severity of disease and the risk of transmission, and prevent potentially lethal complications. Emergence of resistance to first-line therapy like ampicillin, chloramphenicol, and cotrimoxazole including ciprofloxacin among* Salmonella* spp. during the last decades has complicated the situation [1, 2]. For treatment of* Salmonella* infection resistant to these drugs, extended-spectrum cephalosporins (ESCs) are considered as an alternative therapeutic choice. With the increased use of *β*-lactam antibiotics to treat enteric infection,* Salmonella* spp. had acquired resistant to third generation cephalosporin antibiotics in different parts of the world and had been associated with clinical treatment failure [3, 4].

Extended-spectrum beta-lactamases (ESBL) are usually encoded by large plasmids (≥100 kb) that are transferable from strain to strain and between bacterial species [5–7]. Resistance to ESCs is mediated primarily by production of class A ESBLs, which can hydrolyze oxyimino cephalosporins but are not active against cephamycins and carbapenems. The plasmid-encoded CTX-M type ESBLs production was initially identified in 1983 in Germany [8]. The CTX-M family enzymes, which confer high levels of resistance to ESCs, have similar substrate specificities and inhibitor profiles to TEM. The CTX-M type ESBLs have been reported to be found worldwide in different members of Enterobacteriaceae isolated from human and other animal sources [9].

Earlier we have reported the emergence of *bla*_CTX-M_ and *bla*_TEM_ type ESBL producer* S.* Typhi in one-year-old child with recurrent high-grade fever [10]. In recent years there have been several reports indicating the emergence of resistance to *β*-lactam antibiotics among* Salmonella* species [2]. Here, we present *bla*_TEM_ gene mediated ESBL production among* Salmonella* spp. isolated from stool specimen of patients with diarrhea in an urban setting of Bangladesh.

## 2. Material and Methods

As part of a microbiological analysis of stool sample received at Dhaka Treatment Centre of International Centre for Diarrheal Diseases Research, Bangladesh (icddr,b) during 2005–2013, we have analyzed 128,312 fecal specimen following standard microbiological method to identify* Salmonella* spp. Antibiotic susceptibility test was done by Kirby-Bauer disk diffusion test. The putative extended-spectrum beta-lactamase (ESBL) producing strains were tested by the double-disc synergy method and detection of *β*-lactamase genes (*bla*_CTX-M_, *bla*_TEM_, *bla*_SHV_, and *bla*_OXA_ genes) was performed by PCR as describe elsewhere [10].

## 3. Result and Discussion

Overall, the annual incidence of* Salmonella* infection showed a decreasing trend and the proportion came down significantly from 2% in 2005 to 1.0% in 2013 (*P* < 0.001). Of the total* Salmonella* spp. (2120, [1.7%]), nontyphoidal* Salmonella* (NTS) isolates were more frequently isolated than typhoidal* Salmonella* (TS) (72.8% versus 27.2%, *P* < 0.001). Demographic information showed that* Salmonellae* were isolated from patients of all age groups with a maiden age of 4.04 years. The male/female ratio of patients was 1.05. Of the total* Salmonella *positive patients, 51.2% (*n* = 1086) were children aged less than five years. Among the TS serogroups,* S.* Typhi was predominant (404, [65.1%]) followed by* S.* Paratyphi B (139, [22.4%]) and* S.* Paratyphi A (78, [12.6%]). Of the NTS isolates, serogroups C1 strains were more frequently isolated (560, [37.4%]), followed by B (203, [13.5%]), C2 (203, [13.5%]), E (127, [8.5%]), D (94, [6.3%]), G (79, [5.3%]), nontypeable* Salmonella* (54, [3.6%]),* S.* Typhimurium (2, [0.1%]), and A (1, [0.06%]) serogroup. The yearly distribution of* Salmonella* spp. showed distinct seasonality with higher isolation during May to October in each year. Temporal shift was noted in the prevalence of serogroups, seasonality, gender distribution, and resistance pattern between TS and NTS isolates.

**Figure 1:**
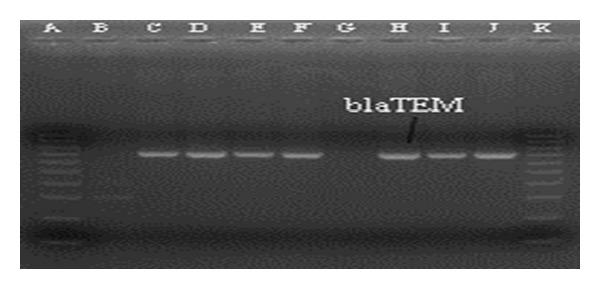
PCR gel electrophoresis of *bla*_TEM_ gene among* Salmonella* strains with AmpCSxtNaCipCro resistance pattern. Lane: A and K, 100 bp ladder; Lane: B, negative control; Lane: C–J, representative* Salmonella* strains.

Around 40% of the* Salmonella* isolates showed resistance to nalidixic acid (Na) followed by 36% to ampicillin (Amp), 20% to cotrimoxazole (Sxt), 4% to ciprofloxacin (Cip), 13% to chloramphenicol (C), and 4% to ceftriaxone (Cro). Interestingly, 32% of the isolates were with reduced susceptibility to ciprofloxacin. Resistance to ≥3 antibiotic classes was more common among TS strains than NTS counterpart (36.6% versus 19.8%, *P* < 0.001). Among the representative* Salmonella* isolates with unusual resistant phenotype (AmpCCipCroNaSxt)^R^, first identified in 2011, *bla*_TEM_ gene was detected in 88% (7/8) of the strains (Figure 1). Interestingly, one* S.* Typhi was positive for both *bla*_TEM_ and *bla*_CTX-M_ genes which is a very rare phenomenon. In our earlier report, we noticed that this same phenomenon was observed in one* S.* Typhi isolated from blood of a typhoid patient [10]. These findings suggest that multi-ESBL producer strains are circulating in Bangladesh. However, to the best of our knowledge, this is the first report of ESBL production among* Salmonella* isolated from stool specimen of diarrheal patients in urban Dhaka, Bangladesh. Detailed molecular characterization including sequencing is necessary for further geno- and subtyping of these strains.

## 4. Conclusion

The study finding reflects the higher prevalence of MAR* Salmonella* spp. among children aged <5 years and *bla*_TEM_ gene mediated ESBL production among* Salmonella* spp. isolated from stool sample of diarrheal patient in urban Bangladesh. Therefore, it is important to establish a surveillance program to understand actual disease burden due to* Salmonella* as well as promote specific and actual line of therapy for Salmonellosis.
